# Psychometric Properties of the Five-Item Ultrashort Oral Health Impact Profile (OHIP5) in the Serbian Cultural Environment: A Cross-Sectional Study

**DOI:** 10.3390/jcm14227909

**Published:** 2025-11-07

**Authors:** Aleksandra Popovac, Jovana Kuzmanović Pfićer, Ivica Stančić, Aleksandra Milić Lemić, Nikola Petričević, Sanja Peršić Kiršić, Asja Čelebić

**Affiliations:** 1 Clinic for Prosthodintics, School of Dental Medicine, University of Belgrade, 11000 Belgrade, Serbia; aleksandra.popovac@stomf.bg.ac.rs (A.P.); ivica.stancic@stomf.bg.ac.rs (I.S.); aleksandra.milic@stomf.bg.ac.rs (A.M.L.); 2 Department for Biomedical Statistics and Informatics, School of Dental Medicine, University of Belgrade, 11000 Belgrade, Serbia; jovana.kuzmanovic@stomf.bg.ac.rs; 3 Department of Removable Prosthodontics, School of Dental Medicine, University of Zagreb, 10000 Zagreb, Croatia; persic@sfzg.hr (S.P.K.); celebic@sfzg.hr (A.Č.)

**Keywords:** oral health-related quality of life, psychometrics, survey, questionnaire

## Abstract

**Background:** Dental patient-centred outcomes are essential in clinical practice and research. To enhance feasibility, Oral Health-Related Quality of Life (OHRQoL) instruments often need to reduce administration time. In Serbia, longer OHIP versions exist (OHIP-14, OHIP-EDENT), but the ultrashort OHIP-5 has not yet been available. Aim: This cross-sectional study aimed to translate, culturally adapt, and evaluate the psychometric properties of the five-item Serbian version of the Oral Health Impact Profile (OHIP5-Srb). **Materials and Methods:** The OHIP5-Srb was translated using a standard forward–backward procedure. Participants were recruited between June and September 2025 using a convenience sampling approach. Psychometric testing—including internal consistency, exploratory (EFA), confirmatory factor analysis (CFA), and convergent and known-groups validity—was conducted on 236 participants (mean age 47.4 years). Test–retest reliability was evaluated in 35 dental students, and responsiveness in 45 patients undergoing dental treatment. **Results:** Cronbach’s alpha was 0.784, indicating adequate internal consistency. Test–retest reliability was excellent (mean ICC = 0.96; all inter-item correlations > 0.20). Convergent validity was supported by a strong negative correlation between OHIP5-Srb summary scores and a single-item measure of overall oral/dental health (Spearman’s rho = −0.861, *p* < 0.01). Known-group validity was confirmed by significant differences between removable denture wearers and individuals with natural teeth (and/or fixed partial dentures), after adjusting for age, and between participants perceiving a need for dental treatment and those who did not. EFA indicated a one-factor structure explaining 55.1% of variance. The one-factor model was confirmed by CFA and showed good fit (χ^2^ = 15.08, df = 5; CFI = 0.97; TLI = 0.94; RMSEA = 0.092; SRMR = 0.04). Responsiveness analysis demonstrated significant decreases in OHIP5-Srb scores following various dental treatments. **Conclusions:** The OHIP5-Srb is unidimensional, reliable, valid, and responsive. Its brevity and robust psychometric properties make it suitable for assessing self-perceived oral health-related quality of life in the Serbian urban population, particularly when minimizing respondent burden is critical.

## 1. Introduction

Many diseases and disorders in the orofacial region affect individuals’ self-perception and quality of life [1]. Therapeutic procedures aimed at improving deteriorated oral health should enhance patients’ overall well-being. Therefore, the assessment of Oral Health-Related Quality of Life (OHRQoL) is essential, as it evaluates oral health and well-being from the individual’s own perspective. Patient-perceived outcomes are crucial in both clinical practice and research settings [2,3]. OHRQoL should be assessed across various fields of dental research and in communities with diverse psychosocial and economic backgrounds. The development of dental health programmes or the implementation of new treatment methods should be based on the measured improvements in OHRQoL resulting from specific therapeutic approaches. OHRQoL gained its deserved importance during the 1980s and 1990s. Multiple-item questionnaires have frequently been used for the assessment of OHRQoL [4,5,6,7,8,9], based on the conceptual framework proposed by Locker [10]. Four generic instruments—the Oral Health Impact Profile (OHIP), the Geriatric Oral Health Assessment Index (GOHAI), the Oral Impacts on Daily Performances (OIDP), and the Subjective Oral Health Status Indicators (SOHSI)—are all derived from Locker’s framework [10].

OHRQoL instruments are culturally sensitive because they reflect perceptions and norms that vary across cultural settings and populations. Among them, the OHIP is the most frequently validated questionnaire for assessing OHRQoL. There are approximately 105 validation studies of the OHIP-14 and 46 validation studies of the OHIP-49 worldwide [9]. Although OHIP-49 and OHIP-14 were originally conceptualized to encompass seven domains, subsequent studies have demonstrated that these instruments consist of only four dimensions: oral function, orofacial pain, orofacial appearance, and psychosocial impact [11,12,13,14].

All multi-item questionnaires impose a burden on both respondents and researchers, which may lead to missing responses. To overcome this limitation, an ultrashort version of the OHIP questionnaire was developed—the OHIP-5—comprising only five items, with at least one item representing each of the four dimensions of oral health [15,16,17]. Recently, the OHIP-5 has been proposed as a core metric instrument for future studies [18,19]. Due to its brevity, it is suitable for population-based studies, evaluations of dental interventions, and various research applications [13,17,18]. The OHIP-5 has been validated in multiple cultural environments and languages [15,16,17,20,21,22,23,24,25,26,27]. Although it contains five items representing the four OHRQoL dimensions, some studies have suggested that the instrument is unidimensional [15,16,17,20,21,22,23,24,25,26,27].

In Serbia, several instruments for assessing OHRQoL have been adapted, including the OHIP-14, OHIP-EDENT, OHRQoL-DW, GOHAI, and OIDP [28,29,30,31,32]. However, given the dynamic nature of contemporary scientific research and the continuous need to evaluate innovative treatments, materials, and methodologies, there remains a clear need for a concise yet valid assessment tool to complement existing instruments in Serbia. Moreover, as patients of all age groups experience reduced attention spans—largely due to the continual increase in screen time—there is a justified concern that longer questionnaires may yield less reliable data or result in incomplete responses [33,34].

When a questionnaire is used in a different cultural context, a rigorous cross-cultural adaptation process is required [35]. Therefore, the aim of the present study was to validate the OHIP-5 questionnaire in the Serbian cultural environment and to explore its dimensionality.

## 2. Materials and Methods

The approval of this cross-sectional study was obtained from the Ethics committee of the School of Dental Medicine, University of Belgrade, (number 36/19, May 2025). The study adhered to ethical principles for medical research involving human subjects according to the Declaration of Helsinki [36].

### 2.1. Translation and Back-Translation

The translation of the OHIP-5 into Serbian was performed from the original English version following the accepted standards of forward–backward translation [37]. One professional translator, together with three dentists with excellent proficiency in English—each of whom had spent at least one year in an English-speaking country after receiving a postgraduate scholarship in dentistry—translated the five OHIP-5 items into the Serbian language. The translated items were then evaluated for lexical accuracy and semantic equivalence by a panel consisting of three general dentists and six specialists from various fields of dental medicine. During this process, several minor cultural adaptations were introduced. In item 2 (“Have you had painful aching in your mouth?”), the initial translation “Da li ste imali bol u ustima?” was modified to “Da li ste imali bolove u ustima ili zubima?”, as the first version referred to a single episode rather than the general occurrence of such symptoms. Similarly, in item 4, the proposed translation “Da li ste osetili da je vaša hrana manje ukusna zbog problema sa zubima, ustima ili vilicom?” was changed to “Da li ste osetili da je hrana koju jedete manje ukusna zbog problema sa zubima, ustima ili vilicom?”. The phrase “your food” was considered less natural in Serbian, while “the food you eat” was deemed more idiomatic and appropriate for everyday use. Although both initial and revised versions were comprehensible, the final ones were preferred for their greater naturalness and familiarity in common speech. Following the panel’s review, the finalized translation was pilot-tested with nine patients currently undergoing dental treatment at the Clinic for Prosthetic Dentistry and the Clinic for Restorative Dentistry, School of Dental Medicine, University of Belgrade. All participants completed the questionnaire, provided feedback through interviews, and confirmed that they fully understood all items without hesitation. Subsequently, a back-translation was performed by two additional Serbian dentists with excellent English proficiency, in collaboration with another professional translator. The back-translated version was compared with the original English instrument and was approved for idiomatic, experiential, and conceptual equivalence by a native English-speaking dentist.

### 2.2. Data Collection, Participants, Inclusion and Exclusion Criteria

To test the psychometric properties of the OHIP5-Srb questionnaire, individuals from different populations were recruited (Table 1). In line with psychometric recommendations, a ratio of ten to twenty participants per item was applied [38,39]. Participants were selected using a convenience sampling approach. Inclusion criteria comprised literate individuals older than 18 years who were willing to participate. Exclusion criteria included individuals whose native language was not Serbian and those with cognitive disorders, dementia, or neurodegenerative diseases, which could be detected through a brief interview during the explanation of the study’s purpose. Participants were recruited from several sources: visitors attending a new movie in one CineStar theatre, individuals accompanying patients in four private dental offices and two university dental clinics in Belgrade, and one private dental office in a rural area near Belgrade. Data collection took place between June and August 2025. The volunteers who distributed the questionnaires briefly explained the study’s aims and objectives to the participants and instructed them to place the completed forms in a designated box if they wished to take part. If it became apparent that a respondent did not understand the instructions, or if any cognitive impairment was suspected, they were not given a questionnaire. Basic literacy was not formally assessed using any testing instrument, as more than 99.37% of individuals aged 10 and above in Serbia are literate [40]. According to the 2022 Census [40], the small proportion of illiterate individuals is predominantly found among the older rural population. However, the volunteers did ask respondents about their educational background, i.e., which school they had completed, and recorded the information as a separate entry to provide additional context regarding literacy levels.

Each participant received a printed version of the OHIP5-Srb questionnaire on a single sheet of paper, along with five additional questions: two regarding age and gender; one asking whether the participant wore a removable denture; one asking whether they believed they needed any oral/dental treatment; and one assessing self-perceived oral health on a five-point Likert scale (1 = completely unsatisfied; 5 = completely satisfied). Participants rated each OHIP5-Srb item using a five-point Likert scale ranging from never to very often (0 = never; 1 = hardly ever; 2 = occasionally; 3 = fairly often; 4 = very often). All questions referred to their experiences during the past seven days [41]. Upon completion, participants were asked to place the completed questionnaire in a designated box nearby.

In total, the general population sample comprised 236 individuals (Table 1), used for testing internal consistency, validity, exploratory factor analysis (EFA), and confirmatory factor analysis (CFA).

### 2.3. Reliability

Reliability was evaluated by testing internal consistency using the standardized Cronbach’s alpha coefficient and inter-item correlation [42]. A Cronbach’s alpha value above 0.70 is considered acceptable, above 0.80 very good, and above 0.90 excellent [42]. Another measure of reliability was test–retest analysis. It was assessed in a convenience sample of 35 dental students aged 20–25 years (Table 1), with a two-week interval between the administrations of the OHIP5-Srb instrument. No changes in the oral cavity or dental interventions were allowed during this period. The intraclass correlation coefficients (ICC(2,1); two-way random-effects model) were calculated based on a one-way repeated-measures analysis of variance. ICC values above 0.80 indicated excellent agreement, while values between 0.60 and 0.80 indicated good agreement [43].

### 2.4. Structural Validity Analysis

In this study, exploratory factor analysis (EFA) and confirmatory factor analysis (CFA) were conducted to assess the structural validity of the questionnaire. Measurement invariance by gender was evaluated to confirm the model’s consistency, including configural, metric, and scalar invariance. The aim was to demonstrate model stability across subgroups and support the robustness of the factor structure.

### 2.5. Convergent Validity

To determine convergent validity, participants rated their oral health status using a single question: “How would you rate your overall oral and dental health?” The responses were given on a 5-point Likert scale (5 = excellent oral health; 4 = very good; 3 = good; 2 = fair; 1 = poor). Spearman’s rank correlation was calculated between the OHIP5-Srb summary scores and the responses to this self-perceived oral health question.

### 2.6. Known-Group Validity

To assess known-group validity, participants indicated whether they were wearing a removable denture (yes/no) and whether they believed they needed any oral/dental treatment (yes/no). It was hypothesized that individuals with their own teeth (or fixed partial dentures) would report better oral/dental health than those wearing removable dentures. Furthermore, it was expected that participants who believed they did not need oral/dental treatment would report better self-perceived oral/dental health than those who believed they required treatment.

### 2.7. Responsiveness

The responsiveness of the Serbian version of the OHIP5 questionnaire was examined to determine its sensitivity in detecting changes in oral health-related quality of life following treatment. Participants completed the OHIP5-Srb before treatment and again between fifteen days and one month after treatment (Table 1). It was hypothesized that OHRQoL would improve after dental treatment, reflected by significantly lower OHIP5-Srb scores.

Among the 45 participants, 15 underwent endodontic root canal treatment due to acute pain (acute pulpitis) and completed the OHIP5-Srb questionnaire before and one month after successful treatment, which was verified by a periapical radiograph. Another 20 participants underwent a teeth-whitening procedure and completed the questionnaire again two weeks after treatment. The remaining ten participants received two implants and Locator-type attachments to support their existing mandibular complete dentures. They completed the OHIP5-Srb questionnaire one month after implant loading, by which time all adjustments and adaptations were expected to be completed [44]. The Wilcoxon signed-rank nonparametric test was used to assess changes in scores. The effect size of treatment was calculated using the formula r = Z/√N, where Z is the standardized test statistic obtained from the nonparametric test and N represents the number of paired observations. According to Cohen’s criteria, r values of approximately 0.10, 0.30, and 0.50 correspond to small, medium, and large effect sizes, respectively [45]. Bland–Altman analysis was also performed [46].

### 2.8. Statistical Analysis

Data analyses were performed using SPSS version 22 (IBM Corp., Armonk, NY, USA), the R (version 4.5.2; lavaan package), and MedCalc Statistical Software (version 13.3.3.0, MedCalc Software Ltd., Ostend, Belgium). Descriptive statistics summarized numeric variables with means, medians, and standard deviations, and categorical data with frequencies and percentages. The distribution of the OHIP5 summary scores and individual-item scores were tested using the one-sample Kolmogorov–Smirnov test. Skewness and Kurtosis were also calculated for summary scores. Categorical variables were compared using Chi-square test (χ^2^), while for numerical ones, Mann–Whitney U and Wilcoxon signed-rank test were used. Analysis of covariance (ANCOVA) was conducted when appropriate to control for potential covariates such as age.

Internal consistency was assessed using Cronbach’s α and Interclass Coefficient (ICC). Concurrent validity was assessed by calculating Spearman’s rank correlation between the OHIP5 Srb scores and self-reported dental/oral health.

Component structure was explored using Exploratory Factor Analysis. The Kaiser–Meyer–Olkin (KMO) measures confirmed the adequacy of the samples, and Bartlett’s Test of Sphericity verified the presence of sufficient correlations among the variables. Factors that met the criteria were those with eigenvalues ≥ 1.0 [47].

In CFA the model fit was assessed using the Chi-square test and the following additional fit indices: Root Mean Square Error of Approximation (RMSEA), Comparative Fit Index (CFI), Tucker–Lewis Index (TLI), and Standardized Root Mean Square Residual (SRMR). Values of RMSEA below 0.08 and CFI and TLI above 0.90 indicated acceptable model fit. SRMR value of 0.08 is taken as acceptable, and a value < 0.05 is considered good (reference Brown, Hu I Kline). Convergent validity was assessed using Composite Reliability (CR) and Average Variance Extracted (AVE), with CR > 0.70 indicating good internal consistency and AVE > 0.50 indicating adequate shared variance. Measurement invariance across gender (male vs. female) was tested through Configural invariance, Metric invariance, and Scalar invariance [48,49,50].

A post hoc power analysis was conducted using G*Power version 3.1.9.7 (Heinrich Heine University Düsseldorf, Düsseldorf, Germany). An F-test was applied within the framework of multiple regression (as an approximation to CFA/factor analysis), with an effect size of f^2^ = 0.15 and a significance level of α = 0.05. The analysis indicated that the sample of 236 participants has high statistical power (power = 0.99), suggesting that it is sufficient to detect medium-sized effects and justifying the conducted analyses.

Floor and ceiling effects were examined by calculating the proportion of participants with the minimum and maximum OHIP—5 scores, respectively. The minimally important difference (MID) was estimated using distribution-based methods, based on 0.5 SD and the standard error of measurement (SEM).

## 3. Results

The respondents’ ages ranged from 18 to 80 years, with a mean age of 47.42 years (standard deviation: 18.57; Table 1). Women comprised 59.7% of the sample, while men accounted for 40.3% (Table 1). According to the volunteers’ notes, 39.8% of participants had completed higher or university-level education, 45.8% had completed secondary education, and 14.4% had completed primary education.

### 3.1. Reliability

Cronbach’s alpha coefficient equaled 0.784, which is considered adequate internal consistency. When one item was deleted, the coefficient varied from 0.65 to 0.75 (Table 2). All inter-item correlations were above the value of 0.20 (Table 3). Due to the exploratory design of this study, formal power calculations were not performed; however, the post hoc obtained power was 99%.

### 3.2. The Test–Retest

**Figure 1:**
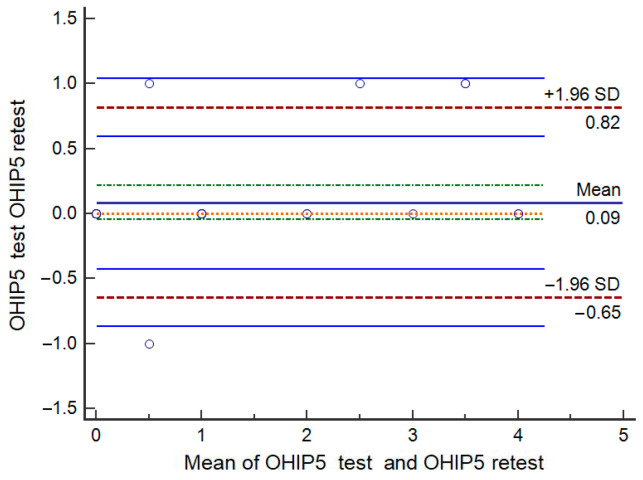
Bland–Altman analysis of test–retest.

The test–retest analysis was conducted as an additional measure of reliability. The ICC(2,1) (two-way random effects model) for the OHIP-5 summary score was 0.955 (95% CI: 0.914–0.977), indicating excellent test–retest agreement. The ICCs for the individual OHIP5-Srb items also demonstrated very good to excellent reliability: Difficulty chewing (ICC(2,1) = 0.900, 95% CI: 0.803–0.977), Painful aching (ICC(2,1) = 0.881, 95% CI: 0.780–0.940), Uncomfortable with appearance (ICC(2,1) = 0.969, 95% CI: 0.940–0.980), Less flavour in food (ICC(2,1) = 0.969, 95% CI: 0.940–0.980), and Difficulty doing usual jobs (ICC(2,1) = 0.810, 95% CI: 0.660–0.900). No significant differences were found between the two test occasions, either for the OHIP5-Srb summary score or for any individual items (*p* > 0.05). Bland–Altman analysis [45] presents a mean difference between test and retest scores of 0.09 (SD = 0.37), indicating no systematic bias. The 95% limits of agreement (−0.64 to 0.82) were narrow, suggesting good agreement between the two measurements (Figure 1).

### 3.3. Validity

#### Exploratory Factor Analysis

The Kaiser–Meyer–Olkin Measure of Sampling Adequacy (0.78) and Bartlett’s test of Sphericity with a significance of <0.0001 were acceptable to perform EFA. Only one factor was extracted. Table 4 shows factor loadings of the OHIP5-Srb (communalities). The results also indicate one-factor nature with an explained variance of 55.12% (Table 5a). Table 5a shows eigenvalues of the EFA, while Table 5b shows communalities.

### 3.4. Confirmatory Factor Analysis

The OHIP5-Srb one-factor model showed a good fit (Table 6) in the sample of 236 subjects (χ^2^ = 15.08, df = 5, *p* = 0.01; CFI = 0.97; TLI = 0.94; RMSEA = 0.092, 90% CI 0.041–0.148; SRMR = 0.04). All OHIP5-Srb questions contribute significantly to the latent factor (*p* < 0.001). Standardized factor loadings (Std.all) were 0.627–0.855 for q1–q5, with q1 having the highest loading (0.855) and q5 the weakest (0.571), which still indicates adequate participation in the oral health construct. The variances of the indicators show that most of the variability of questions q1–q5 is explained by the latent factor, while q5 had a slightly higher residual variance, which is common for short instruments. The RMSEA was slightly higher than ideal (<0.08), which is often the case in models with a small number of indicators.

Convergent validity of the model indicates satisfactory metric characteristics of the instrument. Composite reliability (CR = 0.80) shows good internal consistency of the items, confirming that the OHIP5-Srb items reliably measure a common latent construct. The average variance extracted (AVE = 0.45) was slightly below the recommended value of 0.50. This analysis suggests that the OHIP5-Srb items share moderate common variance and adequately represent the latent construct, although future research could examine ways to improve individual items.

CFA across gender indicated configural invariance and metric invariance, suggesting the factor structure and loadings are comparable between males (N = 95) and females (N = 141) (Table 6). Scalar invariance was largely supported (χ^2^(18) = 35.19, ΔCFI = 0.015, ΔRMSEA = 0.103), allowing comparison of latent means across sex. Factor loadings remained significant in both groups, with standardized loadings ranging from 0.314 to 0.706. Results show that the OHIP-5 scale shows good model fit and measurement invariance across gender.

### 3.5. Known Group Validity

Differences between participants who reported a need for dental treatment and those who did not, as well as between removable denture wearers and individuals with natural teeth or fixed partial dentures, were tested for statistical significance. In both comparisons, the differences were found to be statistically significant, confirming that the instrument effectively discriminates between groups, as predicted (Table 7).

The skewness of the OHIP5 summary score was 1.13 and kurtosis 0.54, allowing the use of parametric ANCOVA to adjust for age, as skewness values between −2 and +2 and kurtosis values between −7 and +7 are considered acceptable [51]. ANCOVA was conducted with the OHIP5 summary score as the dependent variable, removable denture wearing (yes/no) as the factor, and age as a covariate. The effect of age was not significant (F = 1.56, *p* = 0.29), whereas removable denture wearing had a significant effect (F = 62.36, *p* < 0.01). Estimated marginal means were 5.49 (95% CI: 4.87–6.19) for denture wearers and 1.47 (95% CI: 1.00–1.94) for participants with natural teeth.

### 3.6. Responsiveness

As the OHIP5-Srb was expected to be sensitive to different types of dental treatment, three distinct patient groups were selected. A convenience sample of 15 subjects with acute pulpitis received single-visit root canal treatment. After one month, the summary score and all OHIP5-Srb items were significantly reduced compared to pre-treatment scores, except for the item “Uncomfortable about appearance.” All items demonstrated large effect sizes, with the exception of this item. Treatment involving teeth whitening (20 individuals) resulted in a significantly reduced summary score, as well as lower scores for the items “Uncomfortable about appearance” and “Difficulty doing usual jobs.” Large effect sizes were observed for “Uncomfortable about appearance” and for the summary score, while “Difficulty doing usual jobs” showed a medium effect size. The items “Difficulty chewing” and “Painful aching” showed none or only very small effects. The third treatment included in this study was the insertion of two implants with Locator-type attachments in the mandible and the subsequent loading of complete mandibular dentures after four months (10 subjects). One month after treatment, the summary score and all items—except “Uncomfortable about appearance”—showed significantly lower scores, indicating improved OHRQoL and large effect sizes. Table 8 presents the pre- and post-treatment item mean scores (with standard deviations), medians, interquartile ranges, the significance of differences assessed using the Wilcoxon signed-rank test, and the treatment effect sizes for patients who underwent root canal treatment, teeth whitening, or insertion of two implants to support existing mandibular dentures.

### 3.7. Floor and Ceiling Effects, Minimal Clinically Important Difference (MID)

Calculating the floor effect, we found that 32.2% of participants scored 0. No ceiling effect was observed, as no participants reached the maximum possible score of 20. Only 0.4% of participants reached the score of 14. However, using distribution-based methods, the minimally important difference (MID) for the OHIP—5 in our sample was estimated at approximately 1.4–1.5 points.

## 4. Discussion

Procedures for cross-cultural adaptation of any questionnaire developed among different target populations are a critical component of the validation process. Recently, the OHIP5 was recommended as the instrument imposing the least burden on clinicians and respondents when assessing OHRQoL [15,18]. No missing data were observed in this study, which can be attributed to the questionnaire’s brevity and the short time required for completion.

The cultural adaptation and psychometric evaluation were conducted in Belgrade, the largest urban area in Serbia, home to approximately one third of the national population and characterized by diverse social and cultural values. One rural area near Belgrade was also included. Many residents of Belgrade have migrated from other parts of the country, thereby representing a wide range of Serbian population values. Based on the volunteers’ notes, the proportion of participants with secondary or higher education was slightly above the national average reported in the latest Serbian census, but still lower than the proportion reported for the population aged 25 to 34 years in Belgrade [40].

Participants were selected on a voluntary basis, covering a broad age range (18–80 years). The OHIP5-Srb scoring (0–4) was consistent with all other validated OHIP5 versions [15,20,21,22,23,24,25,26,27]. Additionally, responses to a single question assessing self-perceived oral health were provided using a 1–5 scale (1 = worst; 5 = best), consistent with the grading system used in Serbian primary schools, which is familiar to participants. All questions referred to experiences during the preceding seven-day period, as this timeframe has been shown to be more sensitive than a one-month recall period, particularly when testing instrument responsiveness [41].

Overall, the validation of OHIP5-Srb demonstrated favourable psychometric properties. Concurrent validity showed excellent agreement, while known-groups validity confirmed the hypothesis that significant differences would be observed in OHIP5-Srb scores between participants perceiving a need for dental treatment and those who did not, as well as between removable denture wearers and individuals with natural teeth or fixed partial dentures. The skewness and kurtosis of OHIP5 summary scores permitted the use of parametric ANCOVA tests [51], adjusted for age, as participants with dentures were generally older. This analysis further confirmed known-groups validity. Adjustments were made only for summary scores, as both EFA and CFA supported the unidimensional model of the OHIP5-Srb. CFA confirmed a good model fit and satisfactory psychometric properties, with all items significantly contributing to a single latent factor. Measurement invariance across gender indicated comparable performance of the instrument for male and female participants. Test–retest reliability was evaluated using both intraclass correlation coefficients (ICCs) and Bland–Altman analysis. ICC values demonstrated excellent reliability, while the Bland–Altman plot revealed a mean difference of 0.09 (SD = 0.37) between test and retest scores, with 95% limits of agreement ranging from −0.64 to 0.82. These results indicate that the questionnaire provides stable, consistent measurements over time, with no evidence of systematic bias. The narrow limits of agreement support that differences between repeated measurements are small and clinically negligible, confirming suitability for longitudinal or repeated assessments in the Serbian population.

Although OHIP questionnaires with more items (14 or 49) have been shown to measure four dimensions—oral function, orofacial pain, orofacial appearance, and psychosocial impact [8,9,10,11,12,13,14]—the OHIP5 has consistently been found to be unidimensional in various validation studies [15,16,20,21,22,23,25,27]. This unidimensional structure was confirmed for the OHIP5-Srb. Despite its unidimensionality, the OHIP5 includes at least one item representing each of the four original dimensions: “Difficulty chewing” (oral function), “Uncomfortable about appearance” (orofacial aesthetics), “Painful aching” (orofacial pain), and “Less flavour in food” and “Difficulty doing usual jobs” (psychosocial impact).

This multidimensional content is reflected in sensitivity analyses: different dental treatments produced varying effect sizes across items. Root canal treatment yielded the largest effect size in reducing orofacial pain and improving oral function and psychosocial impact. Tooth whitening showed the greatest effect size for orofacial aesthetics (“Uncomfortable about appearance”), as expected. Insertion of two implants to support existing mandibular dentures produced the largest effect size for oral function (chewing), likely due to improved denture stability [52,53,54,55,56,57,58,59,60,61]. The benefits of implant-supported removable dentures and endodontic treatment for acute pulpitis are well documented [52,53,54,55,56,57,58,59,60,61,62,63,64]. These treatments were selected to assess OHIP5-Srb responsiveness, and the study confirmed their benefits through significant improvements in OHRQoL and high effect sizes. However, the study included only three dental treatments to assess responsiveness in small patient groups, which does not encompass all treatment modalities and therefore represents a limitation. Nevertheless, described treatments are commonly performed in dental practices, and the findings offer preliminary insights into the performance and sensitivity of the OHIP5-Srb depending on dental treatments. Broader range of treatments and increased number of patients will be addressed in future studies.

Reliability of the OHIP5-Srb was supported by a satisfactory Cronbach’s alpha coefficient, comparable to English, Swedish, Persian, German, Macedonian, Slovenian, Croatian, and Arabic OHIP5 versions [15,16,20,21,22,23,24,25,26,27]. The coefficient was slightly lower than in longer Serbian OHIP versions (OHIP-14, OHIP-19, and OHIP-49) [29,30], which is consistent with the known relationship between the number of scale items and Cronbach’s alpha [65]. Stability was confirmed via test–retest reliability in dental students, with high ICCs and narrow Bland–Altman limits of agreement, indicating suitability for longitudinal or repeated assessments. Test–retest participants were included only if no dental or oral changes occurred between administrations; dental students were considered reliable in reporting such changes. Minor intraoral changes among lay participants could have gone unnoticed, justifying the selection of dental students for this assessment.

A high proportion of 32.2% of participants with the lowest score zero (floor effect) suggests that the study population has generally good oral health. Also, it implies that the OHIP-5 may have limited sensitivity in distinguishing differences among individuals with very good oral health. No ceiling effect was observed, as none of the participants reached the maximum possible score of 20. Using distribution-based methods, the MID for the OHIP5 in our sample was estimated at approximately 1.4–1.5 points. Thus, while the scale effectively identifies individuals with poorer oral health, its sensitivity to detect minor improvements among those with excellent oral health may be limited. The results are consistent with a recent study, which found that MID values were higher for patients receiving removable dentures than for patients receiving a single- to three-unit fixed partial dentures when no significant change in MID scores was observed (scores were near zero) assessed using either the OHIP-5 or OHIP-14 questionnaires [66].

Certain limitations should be acknowledged. The general population sample was a convenience selection of Serbian adults, excluding groups such as residents from nursing homes, individuals with limited mobility, and those with mental disorders, who are usually not included in questionnaire validation studies, unless specifically targeted. The proportion of participants from rural areas was also relatively small, so the results obtained mostly reflect urban adults within the study’s sampling frame. To minimize respondent burden, socioeconomic data were not included in the questionnaire. Nevertheless, volunteers asked respondents about their educational level and recorded it separately. Future studies should additionally evaluate OHRQoL across different socioeconomic strata. Limited access to dental care remains an issue in rural populations, who are often referred to larger city centres for major dental treatments. Future studies should focus on these groups. Additionally, EFA and CFA were conducted on the same participant sample. Ideally, CFA should be performed on an independent validation sample, but with a total sample size of 236, splitting the data would reduce model stability. Therefore, analyses were conducted on the full sample, consistent with recommendations for studies with limited sample sizes [48,49,50]. Measurement invariance tests across gender (configural, metric, scalar) confirmed model consistency, further supporting factor structure stability. Only three dental treatments in small patient groups were included to provide preliminary responsiveness results. Long-term treatments such as orthodontic therapy [67,68,69] were not included.

Despite these limitations, the OHIP5-Srb demonstrates good psychometric properties and represents a valuable, low-burden instrument suitable for large-scale studies, international comparisons, and the evaluation of new materials and methods, particularly given that patient and therapist perceptions of treatment outcomes may vary across subgroups within the Serbian population. Given the limitation within the sample, the most convenient implementation would be within the urban population where the majority of the studies in Serbia are performed [70,71,72,73,74,75,76,77,78,79,80,81,82,83,84,85,86].

## 5. Conclusions

The ultrashort OHIP5-Srb questionnaire was unidimensional and demonstrated adequate reliability, validity, and responsiveness in the observed sample. These properties make it suitable for assessing self-perceived oral health–related quality of life in the urban Serbian population, particularly in contexts where minimizing time burden is important. Further studies should be addressed to the groups and treatments that were underrepresented in the present study.

## Figures and Tables

**Table 1:** Individuals who took a part in psychometric validation of the OHIP5-Srb together with the research purpose, % of women and their mean age with standard deviations.

Individuals	*n*	(% of Women)	Mean Age ± Standard Deviation	Research Purpose
**General population**	236	59.7%	47.42 (18.57)	Internal consistency, Exploratory factor analysis Convergent validity
**Dental Students**	35	65.7%	22.77 (1.57)	Test–retest reliability
**Patients with treatment needs:**	45	62.2%	42.16 (16.24)	Responsiveness (sensitivity of the OHIP5-Srb to change)
Root canal treatment (acute pulpitis)	15	60.0%	41.4 (8.20)	
Teeth whitening	20	75.0%	30.20 (8.20)	
Mandibular implant-supported overdenture	10	40.0%	67.2 (5.50)	

**Table 2:** Descriptive statistics and Cronbach’s alpha coefficient when one item of the OHIP5-SRB questionnaire was deleted.

OHIP5-Srb						
OHIP5 Item	Mean	Standard Deviation	Median	Interquartile Range	Minimum–Maximum Value	Cronbach’s Alpha If Item Deleted
Difficulty chewing	0.80	1.01	0	1	0–4	0.69
Painful aching	0.52	0.79	0	1	0–3	0.76
Uncomfortable with appearance	0.76	0.97	0	1	0–4	0.74
Less flavour in food	0.54	0.88	0	1	0–3	0.75
Difficulty doing usual jobs	0.18	0.48	0	0	0–3	0.78

**Table 3:** Inter-Item correlation matrix of the OHIP5-SRB.

OHIP5-Srb Inter-Item Correlation Matrix					
Difficulty chewing	1.000				
Painful aching	0.556	1.000			
Uncomfortable about appearance	0.532	0.373	1.000		
Less flavour in food	0.414	0.235	0.430	1.000	
Difficulty doing usual jobs	0.486	0.412	0.314	0.286	1.000

**Table 4:** Factor loadings of the OHIP5-SRB.

	OHIP5-Srb
**Item**	**One Component Extracted**
Difficulty chewing	0.846
Painful aching	0.718
Uncomfortable about appearance	0.736
Less flavour in food	0.630
Difficulty doing usual jobs	0.684

**Table 5:** (**a**) Eigenvalues of the EFA; (**b**) Communalities of the EFA.

(a)						
Total Variance Explained						
Component	Initial Eigenvalues			Extraction Sums of Squared Loadings		
	Total	% of Variance	Cumulative %	Total	% of Variance	Cumulative %
1	2.76	55.12	55.12	2.756	55.12	55.12
2	0.78	15.61	70.72			
3	0.62	12.38	83.10			
4	0.48	9.60	92.70			
5	0.37	7.30	100.00			
(**b**)						
		**Initial**		**Extraction**		
Difficulty chewing		1.00		0.725		
Painful aching		1.00		0.506		
Uncomfortable about appearance		1.00		0.541		
Less flavour in food		1.00		0.522		
Difficulty doing usual jobs		1.00		0.462		

Extraction Method: Principal Component Analysis.

**Table 6:** Confirmatory factor analysis.

Model/Group	N	χ^2^	df	*p*	CFI	TLI	RMSEA	SRMR
Full sample (CFA)	236	15.081	5	0.010 *	0.970	0.940	0.092	0.040
Configural invariance	male/female 95/141	21.625	10	NA	0.964	0.948	0.086	0.057
Metric invariance		26.163	14	0.025 *	0.965	0.948	0.086	0.057
Scalar invariance		35.194	18	0.009 *	0.949	0.943	0.090	0.066

*—statistically significant; CFI—Comparative Fit Index; TLI—Tucker–Lewis Index; MSEA—Root Mean Square Error of Approximation; SRMR—Standardized Root Mean Square Residual.

**Table 7:** Mann–Whitney U tests for known-group validity of the OHIP5-Srb: testing differences between participant groups.

OHIP5-Srb	Self-Perceived Need for a Dental Treatment	N	Mean	Standard Deviation	Median	Interquartile Range	Z	*p*
Difficulty chewing	no	167	0.38	0.59	0	1	−9.72	<0.001 **
	yes	69	1.81	1.09	2	1		
Painful aching	no	167	0.27	0.52	0	0	−7.92	<0.001 **
	yes	69	1.13	0.87	1	1.5		
Uncomfortable about appearance	no	167	0.43	0.74	0	1	−8.54	<0.001 **
	yes	69	1.60	0.99	2	1		
Less flavour in food	no	167	0.32	0.94	0	0	−7.62	<0.001 **
	yes	69	1.19	1.02	1	2		
Difficulty doing usual jobs	no	167	0.05	0.24	0	0	−7.15	<0.001 **
	yes	69	0.51	0.70	0	1		
OHIP5-Srb summary Score	no	167	1.37	1.80	1	2	−10.70	<0.001 **
	yes	69	6.26	2.83	6	5		
**OHIP5-Srb**	**Removable Denture** **Wearing**	**N**	**Mean**	**Standard Deviation**	**Median**	**Interquartile Range**	**Z**	***p***
Difficulty chewing	no	159	0.33	0.54	0	1	−10.38	<0.001 **
	yes	77	1.77	1.05	2	1		
Painful aching	no	159	0.36	0.59	0	1	−4.16	<0.001 **
	yes	77	0.86	0.86	1	2		
Uncomfortable about appearance	no	159	0.49	0.81	0	1	−6.38	<0.001 **
	yes	77	1.32	1.04	1	2		
Less flavour in food	no	159	0.22	0.84	0	0	−9.76	<0.001 **
	yes	77	1.33	1.01	1	1.5		
Difficulty doing usual jobs	no	159	0.08	0.34	0	0	−5.23	<0.001 **
	yes	77	0.39	0.61	0	1		
OHIP5-Srb summary Score	no	159	1.42	1.99	1	2	−9.87	<0.001 **
	yes	77	5.65	3.03	5	3.5		

** = *p* < 0.01; *Z* = *Z* value.

**Table 8:** Descriptive statistics, significance of the differences (before and after treatment) assessed by Wilcoxon signed ranks test together with effect sizes of a treatment.

Acute Pulpitis Treatment (n = 15)	Pre-Treatment OHIP5-Srb			Post-Treatment OHIP5-Srb					
	*x* ± SD	Median	Interquartile Range	x ± SD	Median	Interquartile Range	Z	*p*	Effect Size
Difficulty chewing	2.33 ± 0.62	2	2	0.27 ± 0.46	0	1	−3.53	<0.001 **	0.91
Painful aching	3.13 ± 0.74	3	3	0.47 ± 0.52	0	1	−3.50	<0.001 **	0.90
Uncomfortable about appearance	0.80 ± 0.94	1	1	0.47 ± 0.64	0	1	−1.63	0.10 N.S.	0.42
Less flavour in food	0.47 ± 0.64	0	1	0.00 ± 0.00	0	0	−2.33	0.02 *	0.60
Difficulty doing usual jobs	2.33 ± 0.82	2	2	0.00 ± 0.00	0	0	−3.46	0.001 **	0.89
Summary score	9.07 ± 2.25	10	4	1.20 ± 0.94	1	2	‘3.42	0.001 **	0.88
**Teeth bleaching (n = 20)**	**Pre-Treatment *OHIP5-Srb***			**Post-Treatment *OHIP5-Srb***					
	***x*** ** ± SD**	**Median**	**Interquartile Range**	**x ± SD**	**Median**	**Interquartile Range**	**Z**	***p***	**Effect Size**
Difficulty chewing	0.10 ± 0.31	0	0	0.05 ± 0.22	0	0	−0.10	0.317 N.S.	0.02
Painful aching	0.10 ± 0.31	0	0	0.10 ± 0.31	0	0	0.00	1.000 N.S.	0.00
Uncomfortable about appearance	1.85 ± 0.88	2	2	0.20 ± 0.41	0	1	−3.89	<0.001 **	0.87
Less flavour in food	0.10 ± 0.31	0	0	0.05 ± 0.22	0	0	‘−1.00	0.317 N.S.	0.22
Difficulty doing usual jobs	0.55 ± 0.89	0	1	0.10 ± 0.31	0	0	−2.53	0.011 *	0.50
Summary score	2.70 ± 1.78	3	2.75	0.50 ± 0.69	0	1	−3.76	<0.001 **	0.84
**Two-implants and Locator-type attachments in the mandible (n = 10)**	**Pre-Treatment *OHIP5-Srb***			**Post-Treatment *OHIP5-Srb***					
	***x*** ** ± SD**	***Median***	***Interquartile Range***	**x ± SD**	***Median***	***Interquartile Range***	**Z**	***p***	**Effect Size**
Difficulty chewing	2.40 ± 0.84	2	1	0.60 ± 0.52	1	1	−2.88	0.004 **	0.91
Painful aching	1.60 ± 0.70	1.5	1	0.30 ± 0.48	0	1	−2.74	0.006 **	0.58
Uncomfortable about appearance	0.70 ± 0.67	1	1	0.50 ± 0.71	0	1	−1.40	0.160 N.S.	0.44
Less flavour in food	0.80 ± 0.63	1	1	0.20 ± 0.42	0	0.25	−2.45	0.014 *	0.77
Difficulty doing usual jobs	0.10 ± 1.10	1	2	0.10 ± 0.32	0	0	−2.27	0.023 *	0.72
Summary score	6.60 ± 2.63	6.5	2.5	1.70 ± 1.25	1	2	‘−2.81	0.005 **	0.89

SD = standard deviation; ** = *p* < 0.01; * = *p* < 0.05; N.S. = not significant, x = mean value.

## Data Availability

The original contributions presented in this study are included in the article. Further inquiries can be directed to the corresponding author.

## References

[1] Bennadi D., Reddy C.V. (2013). Oral health related quality of life. J. Int. Soc. Prev. Community Dent..

[2] Argolinha I., Lobo S., Vieira A., Botelho J., Rua J., Mendes J.J., Machado V. (2025). Patient-Reported Outcome Measures in Clinical Practice for Tooth Wear: A Literature Review. J. Clin. Med..

[3] Schmalz G., Denkler C.R., Kottmann T., Rinke S., Ziebolz D. (2021). Oral Health-Related Quality of Life, Oral Conditions, and Risk of Malnutrition in Older German People in Need of Care—A Cross-Sectional Study. J. Clin. Med..

[4] Slade G.D., Spencer A.J. (1994). Development and evaluation of the Oral Health Impact Profile. Community Dent. Health.

[5] Leao A., Sheiham A. (1996). The development of a socio-dental measure of dental impacts on daily living. Community Dent. Health.

[6] Atchison K.A., Dolan T.A. (1990). Development of the Geriatric Oral Health Assessment Index. J. Dent. Educ..

[7] Slade G.D. (1997). Derivation and validation of a short-form oral health impact profile. Community Dent. Oral Epidemiol..

[8] Cushing A.M., Sheiham A., Maizels J. (1986). Developing socio-dental indicators—The social impact of dental disease. Community Dent. Health.

[9] Riva F., Seoane M., Reichenheim M.E., Tsakos G., Celeste R.K. (2022). Adult oral health-related quality of life instruments: A systematic review. Community Dent. Oral Epidemiol..

[10] Locker D. (1988). Measuring oral health: A conceptual framework. Community Dent. Health.

[11] John M.T., Reißmann D.R., Feuerstahler L., Waller N., Baba K., Larsson P., Čelebić A., Szabo G., Rener-Sitar K. (2014). Factor analyses of the Oral Health Impact Profile—Overview and studied population. J. Prosthodont. Res..

[12] John M.T., Reissmann D.R., Feuerstahler L., Waller N., Baba K., Larsson P., Celebić A., Szabo G., Rener-Sitar K. (2014). Exploratory factor analysis of the Oral Health Impact Profile. J. Oral Rehabil..

[13] John M.T., Reissmann D.R., Čelebić A., Baba K., Kende D., Larsson P., Rener-Sitar K. (2016). Integration of oral health-related quality of life instruments. J. Dent..

[14] John M.T., Sekulić S., Bekes K., Al-Harthy M.H., Michelotti A., Reissmann D.R., Nikolovska J., Sanivarapu S., Lawal F.B., List T. (2020). Why Patients Visit Dentists—A Study in All World Health Organization Regions. J. Evid.-Based Dent. Pract..

[15] Rener-Sitar K., Čelebić A., Križaj M., Petričević N. (2025). Psychometric Validation of the Ultrashort Slovenian (OHIP-SVN5) and Croatian (OHIP-CRO5) Oral Health Impact Profile Questionnaires. Zdr. Varst..

[16] John M.T., Miglioretti D.L., LeResche L., Koepsell T.D., Hujoel P., Micheelis W. (2006). German short forms of the Oral Health Impact Profile. Community Dent. Oral Epidemiol..

[17] Naik A., John M.T., Kohli N., Self K., Flynn P. (2016). Validation of the English-language version of 5-item Oral Health Impact Profile. J. Prosthodont. Res..

[18] John M.T. (2022). Standardization of dental patient-reported outcomes measurement using OHIP-5—Validation of “Recommendations for Use and Scoring of Oral Health Impact Profile Versions”. J. Evid.-Based Dent. Pract..

[19] Reissmann D.R. (2021). Methodological considerations when measuring oral health-related quality of life. J. Oral Rehabil..

[20] Alhajj M.N., Halboub E., Khalifa N., Amran A.G., Reissmann D.R., Abdullah A.G., Assad M., Al-Basmi A.A., Al-Ghabri F.A. (2018). Translation and validation of the Arabic version of the 5-item Oral Health Impact Profile: OHIP5-Ar. Health Qual. Life Outcomes.

[21] Lü H., He F.M. (2020). Reliability and validity of the Chinese version of the 5-item oral health impact profile. Hua Xi Kou Qiang Yi Xue Za Zhi.

[22] Nazeri A.M., Nakhaee N., Navabi N. (2020). Validation of an Ultrashort Persian Version of Oral Health Impact Profile (OHIP-5) Questionnaire. Pesqui. Bras. Odontopediatria Clín. Integr..

[23] Simancas-Pallares M., John M.T., Enstad C., Lenton P. (2020). The Spanish Language 5-Item Oral Health Impact Profile. Int. Dent. J..

[24] Larsson P., John M.T., Hakeberg M., Nilner K., List T. (2014). General population norms of the Swedish short forms of oral health impact profile. J. Oral Rehabil..

[25] León S., Correa-Beltrán G., De Marchi R.J., Giacaman R.A. (2017). Ultra-short version of the oral health impact profile in elderly Chileans. Geriatr. Gerontol. Int..

[26] Elenčevski S., Čelebić A., Popovac A., Apostolska S., Nikolovska J., Stančić I. (2025). Psychometric Validation of the Macedonian-Language Version of the Ultrashort Five-Item Oral Health Impact Profile in the North Macedonian Population (OHIP5-MAC). Medicina.

[27] Solanke C., John M.T., Ebel M., Altner S., Bekes K. (2024). OHIP-5 for School-Aged Children. J. Evid.-Based Dent. Pract..

[28] Stancić I., Sojić L.T., Jelenković A. (2009). Adaptation of Oral Health Impact Profile (OHIP-14) index for measuring impact of oral health on quality of life in elderly to Serbian language. Vojnosanit. Pregl..

[29] Čelebić A., Stančić I., Kovačić I., Popovac A., Topić J., Mehulić K., Elenčevski S., Peršić S. (2020). Psychometric Characteristics of the Croatian and the Serbian Versions of the Oral Health Impact Profile for Edentulous Subjects, with a Pilot Study on the Dimensionality. Zdr. Varst..

[30] Jovanović M., Janković S., Milojević Samanović A., Gojak R., Raičević B., Erić J., Milosavljević M. (2024). A New Scale for Rating Oral Health-Related Quality of Life in Denture Wearers. Oral Health Prev. Dent..

[31] Popović Z., Gajić I., Obradović-Djuricić K., Milosević D.P. (2015). Introduction to Verification of the GOHAI Instrument for Measuring the Oral Health-Related Quality of Life in Patients with Dentures Using the Serbian Preliminary Version—A Pilot Study. Vojnosanit. Pregl..

[32] Stancić I., Kulić J., Tihacek-Sojić L., Stojanović Z. (2012). Applicability of a Serbian Version of the “Oral Impacts on Daily Performance (OIDP)” Index—Assessment of Oral Health-Related Quality of Life. Vojnosanit. Pregl..

[33] Harvey D.L., Milton K., Jones A.P., Atkin A.J. (2022). International trends in screen-based behaviours from 2012 to 2019. Prev. Med..

[34] Shalash R.J., Arumugam A., Qadah R.M., Al-Sharman A. (2024). Night screen time is associated with cognitive function in healthy young adults: A cross-sectional study. J. Multidiscip. Healthc..

[35] Beaton D.E., Bombardier C., Guillemin F., Ferraz M.B. (2000). Guidelines for the Process of Cross-Cultural Adaptation of Self-Report Measures. Spine.

[36] Ehni H.J., Wiesing U. (2024). The Declaration of Helsinki in Bioethics Literature since the Last Revision in 2013. Bioethics.

[37] Guillemin F., Bombardier C., Beaton D. (1993). Cross-Cultural Adaptation of Health-Related Quality of Life Measures: Literature Review and Proposed Guidelines. J. Clin. Epidemiol..

[38] Mokkink L.B., Terwee C.B., Knol D.L., Stratford P.W., Alonso J., Patrick D.L., Bouter L.M., de Vet H.C. (2010). The COSMIN checklist for evaluating the methodological quality of studies on measurement properties: A clarification of its content. BMC Med. Res. Methodol..

[39] Mokkink L.B., Elsman E.B.M., Terwee C.B. (2024). COSMIN guideline for systematic reviews of patient-reported outcome measures version 2.0. Qual. Life Res..

[40] Vreme. Census Results: Increased Number of Educated People in Serbia. https://vreme.com/en/vesti/rezultati-popisa-povecan-broj-obrazovanih-u-srbiji/.

[41] Waller N., John M.T., Feuerstahler L., Baba K., Larsson P., Peršić S., Kende D., Reißmann D.R., Rener-Sitar K. (2016). A 7-Day Recall Period for a Clinical Application of the Oral Health Impact Profile Questionnaire. Clin. Oral Investig..

[42] Cronbach L.J. (1951). Coefficient Alpha and the Internal Structure of Tests. Psychometrika.

[43] Shrout P.E., Fleiss J.L. (1979). Intraclass Correlations: Uses in Assessing Rater Reliability. Psychol. Bull..

[44] Topić J., Poljak-Guberina R., Persic-Kirsic S., Kovacic I., Petricevic N., Popovac A., Čelebić A. (2022). Adaptation to New Dentures and 5 Years of Clinical Use: A Comparison between Complete Denture and Mini-Implant Mandibular Overdenture Patients Based on Oral Health-Related Quality of Life (OHRQoL) and Orofacial Esthetics. Acta Stomatol. Croat..

[45] Cohen J. (1988). Statistical Power Analysis for the Behavioral Sciences.

[46] Bland J.M., Altman D.G. (1986). Statistical methods for assessing agreement between two methods of clinical measurement. Lancet.

[47] Tabachnick B.G., Fidell L.S. (2019). Using Multivariate Statistics.

[48] Brown T.A. (2006). Confirmatory Factor Analysis for Applied Research.

[49] Hu L.T., Bentler P.M. (1999). Cutoff criteria for fit indexes in covariance structure analysis: Conventional criteria versus new alternatives. Struct. Equ. Model..

[50] Kline R.B. (2016). Principles and Practice of Structural Equation Modeling.

[51] Cain M.K., Zhang Z., Yuan K.H. (2017). Univariate and multivariate skewness and kurtosis for measuring nonnormality: Prevalence, influence and estimation. Behav. Res. Methods.

[52] Singh S., Mishra S.K., Chowdhary R. (2023). Patient Satisfaction and Crestal Bone Changes with One-Piece and Two-Piece Single Implant-Retained Mandibular Overdenture: A Randomized Controlled Clinical Study. J. Prosthodont. Res..

[53] Schimmel M., Araujo M., Abou-Ayash S., Buser R., Ebenezer S., Fonseca M., Heitz-Mayfield L.J., Holtzman L.P., Kamnoedboon P., Levine R. (2023). Group 4 ITI Consensus Report: Patient Benefits Following Implant Treatment in Partially and Fully Edentulous Patients. Clin. Oral Implants Res..

[54] Elsyad M.A., Tella E.A.E.S., Mohamed S.S., Mahrous A.I. (2022). Within-Patient Evaluation of Chewing Efficiency and Maximum Bite Force of Conventional Dentures, Fixed Prostheses, and Milled Bar Overdentures Used for All-on-4 Implant Rehabilitation of Atrophied Mandibular Ridges: A Short-Term Randomized Trial. Clin. Implant Dent. Relat. Res..

[55] Yeung A.W.K., Leung W.K. (2023). Functional Neuroplasticity of Adults with Partial or Complete Denture Rehabilitation with or without Implants: Evidence from fMRI Studies. Nutrients.

[56] Dakhilalian M., Rismanchian M., Fazel A., Basiri K., Azadeh H., Mahmoodi M., Fayazi S., Sadr-Eshkvari P. (2014). Conventional versus Implant-Retained Overlay Dentures: A Pilot Study of Masseter and Anterior Temporalis Electromyography. J. Oral Implantol..

[57] Čelebić A., Kovačić I., Petričević N., Alhajj M.N., Topić J., Junaković L., Peršić-Kiršić S. (2023). Clinical Outcomes of Three versus Four Mini-Implants Retaining Mandibular Overdenture: A 5-Year Randomized Clinical Trial. Medicina.

[58] Peršić S., Ćelić R., Vojvodić D., Petričević N., Kranjčić J., Zlatarić D.K., Čelebić A. (2016). Oral Health-Related Quality of Life in Different Types of Mandibular Implant Overdentures in Function Longer Than 3 Years. Int. J. Prosthodont..

[59] Schuster A.J., Salybi S.R.B., Possebon A.P.D.R., Schinestsck A.R., Faot F. (2024). 3-Year Bone Remodeling in Mandibular Overdenture Wearers: Results from an RCT Comparing Immediate vs. Conventional Loading Using CBCT. Int. J. Prosthodont..

[60] Fonteyne E., Matthys C., Bruneel L., Becue L., De Bruyn H., Van Lierde K. (2021). Articulation, Oral Function, and Quality of Life in Patients Treated with Implant Overdentures in the Mandible: A Prospective Study. Clin. Implant Dent. Relat. Res..

[61] Petricevic N., Celebic A., Puljic D., Milat O., Divjak A., Kovacic I. (2024). Effects of loading forces, loading positions, and splinting of two, three, or four Ti-Zr (Roxolid^®^) mini-implants supporting the mandibular overdentures on peri-implant and posterior edentulous area strains. J. Funct. Biomater..

[62] Alsultan M., Srivastava S., Javed M.Q., Khan M., Ulfat H. (2023). Influence of Root Canal Treatment on Oral-Health-Related Quality of Life (OHRQoL) in Saudi Patients: A Cross-Sectional Study. Cureus.

[63] Edwards D., Rasaiah S., Hamzah Ahmed S., Breckons M., Stone S.J., Currie C.C., Durham J., Whitworth J. (2023). The Financial and Quality of Life Impact of Urgent Dental Presentations: A Cross-Sectional Study. Int. Endod. J..

[64] Patel N., Khan I., Jarad F., Zavattini A., Koller G., Pimentel T., Mahmood K., Mannocci F. (2025). The Short-Term Postoperative Pain and Impact upon Quality of Life of Pulpotomy and Root Canal Treatment, in Teeth with Symptoms of Irreversible Pulpitis: A Randomized Controlled Clinical Trial. Int. Endod. J..

[65] Cortina J.M. (1993). What Is Coefficient Alpha? An Examination of Theory and Applications. J. Appl. Psychol..

[66] Limpuangthip N., Wannakhao P. (2025). Validation, measurement properties, and minimal clinically important difference of short-version oral health impact profile (OHIP-5) by type of prosthodontic treatment: A prospective cohort study. BMC Oral Health.

[67] Alhamwi A.M., Burhan A.S., Idris M.I., Nawaya F.R. (2024). Duration of Orthodontic Treatment with Clear Aligners versus Fixed Appliances in Crowding Cases: A Systematic Review. Clin. Oral Investig..

[68] Brugnami F., Meuli S., Ventura V., Gentile D. (2025). Long-Term Stability and Histologic Evaluation of Orthodontically Driven Osteogenesis (ODO): A Preliminary Retrospective Study. J. Clin. Med..

[69] Karvelas N., Samandara A., Dragomir B.R., Chehab A., Panaite T., Romanec C., Papadopoulos M.A., Zetu I.N. (2025). Evaluation of Maxillary Molar Distalization Supported by Mini-Implants with the Advanced Molar Distalization Appliance (amda^®^): Preliminary Results of a Prospective Clinical Trial. J. Clin. Med..

[70] Llaneza A.J., Stone K.A., Seward J. (2025). Weaving Oral Health Provider Perspectives to Guide Future Dental Therapy Advocacy and Implementation Efforts. Front. Public Health.

[71] Atmeh A., Al-Hadi Hamasha A. (2020). Outcome Assessment of Non-Surgical Root Canal Treatment by Patients: What Factors Can Influence Their Evaluation?. Br. Dent. J..

[72] Lamprecht R., Struppek J., Heydecke G., Reissmann D.R. (2020). Patients’ Criteria for Choosing a Dentist: Comparison between a University-Based Setting and Private Dental Practices. J. Oral Rehabil..

[73] Gotfredsen K. (2023). Patient-Reported Outcomes for Bone Regenerative Procedures. Periodontol. 2000.

[74] Alajlan A., Alhoumaidan A., Ettesh A., Doumani M. (2019). Assessing Knowledge and Attitude of Dental Patients Regarding the Use of Dental Implants: A Survey-Based Research. Int. J. Dent..

[75] Mocelin R.C., Penteado M.M., Pierre F.Z., Saraiva A.C.V., Uemura E.S., da Silva J.M.F. (2021). Assessment of Patient and Dentist Preference between Conventional and Digital Diagnostic Waxing. Int. J. Esthet. Dent..

[76] Jafarpour D., Feine J.S., Morris M., Souza R.F. (2024). Patient-Reported Outcomes and Clinical Performance of CAD/CAM Removable Dentures: A Scoping Review. Int. J. Prosthodont..

[77] Gullberg J., Lindh C., Axtelius B., Horner K., Devlin H., Povlsen L. (2020). Osteoporosis Risk Assessment in Primary Dental Care—The Attitudes of Swedish Dentists, Patients and Medical Specialists. Gerodontology.

[78] Yap A.U., Marpaung C. (2024). Psychologic Factors in Temporomandibular Disorders and Somatization: A Multidimensional Analysis of Personality, Coping, and Distress among Young Adults. Int. J. Prosthodont..

[79] Zhao Y., Surdu S., Langelier M. (2024). Safety Net Patients’ Satisfaction with Oral Health Services by Provider Type and Intent to Return for More Care. J. Public Health Dent..

[80] Čelebić A., Valentić-Peruzović M., Stipetić J., Delić Z., Staničić T., Ibrahimagić L. (2000). The Patient’s and the Therapist’s Evaluation of Complete Denture Therapy. Coll. Antropol..

[81] Seager L., Shah J., Burke T., Khambay B. (2021). A Study of Smile Aesthetic Perception among Dental Professionals, Patients and Parents towards Impacted Maxillary Canine Treatment Options. J. Orthod..

[82] Azarpazhooh A., Dao T., Ungar W.J., Da Costa J., Figueiredo R., Krahn M., Friedman S. (2016). Patients’ Values Related to Treatment Options for Teeth with Apical Periodontitis. J. Endod..

[83] Golea A., Tat R.M., Vesa Ș.C., Mitrofan D., Boeriu C., Rotaru L.T., Cimpoeșu D.C., Nica S., Petrică A., Puticiu M. (2025). Thirty Years of Emergency Medicine in Romania—A Bridge Between the Behavior of Emergency Department Professionals and the Health System Management Strategy: A Survey Study. J. Clin. Med..

[84] Mendes J.J. (2025). Advancing the Understanding of Oral Health Through Multidisciplinary and Translational Perspectives: Insights from a Special Issue of the *Journal of Clinical Medicine*. J. Clin. Med..

[85] Joshi A.Y., Bansal P., Hong S., Bingemann T.A. (2024). The Role of Patient Satisfaction Scores in Clinical Care and Physician Wellness. J. Allergy Clin. Immunol. Pract..

[86] Diamond-Brown L. (2016). The Doctor–Patient Relationship as a Toolkit for Uncertain Clinical Decisions. Soc. Sci. Med..

